# Revival of Symphyseotomy

**Published:** 1894-10

**Authors:** W. J. Sinclair

**Affiliations:** M. D., Manchester, Eng.


### REVIVAL OF SYMPHYSEOTOMY.

By W. J. SINCLAIR, M. D., Manchester, Eng.

THE literature of this subject has in recent years become so voluminous that it would be impossible to find space to do more than summarize the accounts of the most striking work done in Italy, France and Germany, where the operation has been most frequently performed, and has found the most prominent supporters.

*Historical.*—The operation was first proposed by Sigault, of Dijon, in 1768, and first performed at Naples by Domenico Ferrara in 1774. Ever since then there have been champions of the operation in Naples,[1] notably Amantea and Galbiati, and in more recent times Spinelli, Caruso and Morisani. The last of these called attention to his operative work for the first time in 1863, and his contribution of facts and opinions on symphyseotomy was perhaps the most remarkable feature of the Gynecological Section's Proceedings at the recent Congress at Rome. Throughout the rest of Europe the operation was entirely abandoned as too dangerous. This result was largely owing to the discussion which took place between Sigault and Baudelocque. In this discussion Baudelocque appeared to bring forward unanswerable arguments drawn from his experiments upon the cadaver, especially proving the absence of increase in the conjugata vera and the occurrence of fracture of the sacro-iliac joints as results of the operation; and though his conclusions were shown later to be erroneous, they were accepted at the time. It was not until great advances had been made towards exact pelvimetry, and antiseptics had been introduced into obstetric surgery, that the results obtained by the Italians were sufficiently encouraging to attract favorable attention in France and Germany, and, curiously enough, it was at the

1. Corradi. History of Obstetrics in Italy during the 18th Century.

Clinique Baudelocque in Paris that the most strenuous advocates of symphyseotomy were found.

*Symphyseotomy in Germany.*—The Transactions of the German Gynecological Society at its meeting at Breslau in May, 1893, will perhaps best serve as the basis of a review of the German material up to that date.

Zweifel, of Leipzig, gave an account of his experience, referring to previous papers of his own and of others on the same subject, and frankly admitting that his opinion on the subject had undergone a complete change. He mentioned fourteen cases of symphyseotomy occurring in his clinic. All the fourteen women recovered completely, without any unfitness for exertion. Two of the children were stillborn, or died immediately after birth. He found it impracticable to follow the example of the Neapolitan school, and wait for spontaneous delivery after the cutting portion of the operation. He had to apply the forceps in ten of the cases, but always with the head low in the pelvis. The weakening of the fetal heart-sounds was in every case the indication for the use of the forceps, and in six of the cases coiling of the cord round the neck of the fetus was found to be the explanation of the indications.

As to the *technique* of the operation, Zweifel calls attention to the following details. The incision through the skin gives rise to smart hemorrhage, which can be readily stopped. The introduction of the finger behind the symphysis and the protection of the knife in cutting through the cartilage from above downwards are easily effected. Only in one case had he to use the chain-saw, owing to difficulty in completing the incision through the symphysis. Difficulties may arise here, not from ossification, but from obliquity in the direction of the symphysis or irregularity in the form of the pubic bones. When the cartilage is cut through, the pubic bones separate about a quarter of an inch, and when the last fibers of the ligamentum arcuatum give way, the distance between the bones amounts to a finger's breadth. Then comes on very considerable hemorrhage, which is difficult to stop. It is best arrested by the introduction of a tampon with counter-pressure from the vagina. When this has been done, the patient may be put back to bed and labor allowed to proceed for twelve or fourteen hours. In many cases the os is not fully dilated, although the membranes may have ruptured at the time of the incision. When the head is passing through the narrow portion of the pelvis, the pubic bones sometimes separate to the extent of $2\frac{1}{2}$ to $2\frac{3}{4}$ inches. In such cases there is always fresh hemorrhage and the need for additional *tamponnade* to arrest it.

After the completion of parturition, another set of circumstances demand attention. Zweifel says that the old accoucheurs used to fix a serviette round the pelvis, and healing took place. He himself had to resort to suture of the bones in eleven cases. He describes the difficulties in the way. He used silver wire and it cut through ; he separated the periosteum with a raspatory, and took a larger hold of the bone ; and he finally succeeded in bringing the separated surfaces of the symphysis together. He says this proceeding is not difficult, but recommends that it should not be attempted in private practice. It may be sufficient to follow the Italian method of suturing the fascia, and so turning in the feet when the legs are extended as to diminish dragging upon the symphysis. In order to keep the pelvis still and immovable, a special girdle was used at first, but in later cases strips of plaster fixed round the pelvis were found to be as efficient and more comfortable. In three cases in which silver wire was used, incontinence of urine came on, and required treatment later. In Zweifel's cases the ability to walk soon returned. There was a slight increase in every case in the transverse diameter of the pelvis.

The puerperium was favorable throughout in only three of the cases, and five of the number were ill, with high temperature, for a long time.

Attention is called to the extensive lacerations of the maternal soft parts, which may be as serious as the injuries to the sacro-iliac joint. Such lacerations occurred in Zweifel's cases once spontaneously and three times during forceps delivery. Three of the women were primiparæ. Later experience has led many operators to the conclusion that symphyseotomy should not be performed upon any woman during her first labor.

At the same congress Döderlein, also of Leipzig, described and illustrated his observations on certain questions bearing on the operation. He set himself, first, to answer the question, What is the condition of the sacro-iliac synchondrosis when the pubic bones are separated during the operation? The second question to be answered was, Is the amount of room in the pelvis gained by the operation a sufficient advantage to place against the dangers and difficulties of the operation? On the first head he found, contrary to the accepted traditional views of Baudelocque, that in the pregnant woman at full term the sacro-iliac joint does not fracture; there is only a partial tearing of the capsule of the joint and especially of the anterior sacro-iliac ligament. The joint is opened and the fluid in the joint escapes; if small vessels are torn there may even be an extravasation of blood into the joint. As soon, however, as the pelvis is restored to its proper shape on the completion of labor, the injuries are quickly and completely healed if no infection has occurred. On the question of increased space in the pelvis Döderlein proved by experiment that when the pubic bones were separated six centimeters, there was an increase in the inlet equivalent to fifty square centimeters, and when the symphysis was separated eight centimeters there was an increased area of the inlet equivalent to sixty-six square centimeters.

Leopold, of Dresden, had performed the operation four times. He thought it was not an operation for general practice, and was not likely to become so. The upper limit for the conjugata vera is 8cm. ($3\frac{1}{8}$in.), and the lower limit 6cm. ($2\frac{3}{8}$in.) Labor should be allowed to proceed until the os is completely dilated, and then the whole process should be completed at once by the application of the forceps. The greatest possible care must be exercised to prevent laceration of the vagina. Primiparæ should never be subjected to the operation.

The discussion was continued by Chrobak and Schauta, of Vienna; Olshausen, of Berlin; Fehling, of Basel; and others who had done the operation very seldom or not at all. It brought out differences of opinion on various points, such as the relative danger of injuries to the bones and to the soft parts, the need for suture of the bony parts (Knochen-naht), the relative frequency of pyrexia and symptoms of sepsis after operation, and to what extent symphyseotomy would do away with the necessity for perforation of the living fetus.

Such was the state of information and opinion among German obstetricians little more than a year ago.

In the discussion on symphyseotomy, which took place at the International Medical Congress in March last, Leopold and Zweifel among others took part, and the former said that it was his duty to explain the position in the controversy occupied by German obstetricians. He and his colleagues, however, brought out the fact that they had not in any way changed their views on controverted points. Leopold still spoke in favor of premature labor practised not earlier than the thirty-fourth week, and Zweifel advocated, as formerly, delay after the cutting portion of the operation, so as to avoid the application of the forceps. His cases had now increased to twenty-three, all the mothers were alive, and twenty-one of the children. There had been no incontinence of urine, no fistula, no serious puerperal affection, but in three of the women walking continued for a long time to be difficult.

Sänger, of Leipzig, joined in the discussion to compare the operation with Cesarean section. He said his mortality for twelve cases was *nil.* In general the mortality of the two operations differs little. If we compare the time occupied by the two operations, we find that Cesarean section takes about an hour, and requires no after-treatment, whereas symphyseotomy, according to Zweifel's method, demands about a day, and the after-treatment continues for about a year. Symphyseotomy, in Sänger's opinion, ought to be more limited in scope, and that of the Cesarean operation extended.

*Symphyseotomy in France.*—The revival of symphyseotomy in France chiefly depends upon the clinical work of Pinard. This clinical work is explained and justified by the anatomical and experimental studies of Professor Farabeuf[1] and of Dr. Varnier.

Up to the time of delivering his address on symphyseotomy, at Rome, in March last, Pinard had performed the operation thirty-six times, with the result that two mothers and four children died. No accident had occurred in any case during the incision of the symphysis and enlargement of the pelvis. Hemorrhage had always been stopped by using a sponge as tampon. During the extraction of the fetus in primiparæ, laceration of the anterior vaginal wall occurred five times. The tears were not extensive, and gave rise to little trouble. The practice of preliminary dilatation by means of Champetier's bag rendered laceration a rare occurrence. Incontinence of urine continued for a considerable time in one case. In all the cases consolidation of pelvis was complete by about the twentieth day. The after-history, in a considerable number of the cases, showed that the women were engaged at laborious occupations, and did not suffer in any way. One of the women became pregnant about three months after undergoing the operation, and went through normal spontaneous parturition at full term in the *clinique.*

1. Possibilité et moyens de traiter scientifiquement la dystocie du détroit supérieur retréci. *Annales de Gynécologie*, May-June, 1894.

Pinard gives a table showing a comparison between the results obtained in cases of contracted pelvis by the induction of premature labor and by symphyseotomy. In sixty-four cases of the former, two mothers and thirty children were lost, whereas in thirty-six of the latter, two mothers and four children were lost. The author proceeds to give a *résumé* of the anatomical and experimental researches of Farnbeuf. He begins with the remark, "*Ces notions anatomiques sont ennuyeuses.*" With this opinion most readers will agree. The summary itself is long, and incapable of further condensation. Pinard then gives a carefully-detailed and illustrated account of his method of operating. He "indicates the method by which the difficulties and dangers of the operative proceedings are to be suppressed."

He finally formulates his conclusions as follows:

1. Symphyseotomy or pubiotomy performed antiseptically is not a dangerous operation.

2. In order to be useful it ought to be complete, and the preliminary separation of the pubic bones ought to be in proportion to the contraction of the pelvis.

3. The operation should be performed only on those cases in which examination and measurement of the pelvis show that separation of the pubic bones to the extent of $2\frac{3}{4}$ inches will permit the passage of the fetal head at term.

4. Separation of the pubic bones exceeding $2\frac{3}{4}$ inches, being capable of producing lesions of the soft parts, should be proscribed.

5. When the contraction of the pelvis is such that separation of the pubic bones to the extent of $2\frac{3}{4}$ inches will not permit of the passage of the head of the fetus, recourse should be had to Cesarean section (Porro's operation).

6. In case of oblique ovular pelvis, with synostosis of one of the sacro-iliac articulations, when the contraction does not permit of spontaneous delivery, it is best to perform the operation of Farabeuf, that is, ischio-pubiotomy.

He adds the following "general conclusions":

1. Embryotomy, cephalotripsy, and waiting for the death of the fetus ought to be forever proscribed.

2. Operative enlargement of the pelvis, conducted according to the rules laid down, ought to lead to the abandonment of (*a*) the induction of premature labor; and (*b*) every operative proceeding having for its object to make the fetal head struggle against osseous resistance which cannot be overcome by the uterine contractions.

*Symphyseotomy in Italy.*—The work of the German and French obstetricians in symphyseotomy is a mere reflection of that done in Italy, and more especially of the work of Morisani, of Naples. Morisani's first publication, On the Contractions of the Pelvis and the Indications which Appear at the Time of Parturition, attracted considerable attention to symphyseotomy. It appeared in 1863, and since then numerous papers have conveyed to the medical profession a knowledge of his clinical work and of his opinions. He has also been aided in his advocacy by able colleagues and disciples, who have been active clinical workers and diligent contributors to the literature of the subject. One of the most useful of these contributions was that of Spinelli, *Sulla Sinfisiotomia, Annotazioni Storiche, Critiche e Bibliografiche*, which ran through several numbers of the *Annali di Ostetricia e Ginecologia* in 1892. Among the numerous addresses and papers of Morisani, one of the most important was a contribution to the same journal in January, 1893, *Per la Sinfisiotomia*, in which he gave the statistics and details of fifty-five cases. And now, quite recently, at the International Congress, his address on Symphyseotomy placed before the profession his most mature experience and reflections.

Symphyseotomy is now rendered a justifiable operation by our clinical experience. We are enabled by it to extract a well-developed child at term through a contracted pelvis with a conjugate diameter of 6.7 cm. to 8.8 cm. ($2\frac{5}{8}$ to $3\frac{1}{2}$ inches). The operation is contra-indicated when the fetus is dead, or even very feeble. The time for operation is when the pains are in full force and the dilatation of the os is well advanced. If it be objected that with an antero-posterior diameter of $3\frac{3}{8}$ inches spontaneous birth is possible, or the forceps may readily complete the parturition, it may be answered that a favorable termination is only exceptional, and that the use of the forceps in such circumstances is "masked cephalotripsy," with this difference, that the ordinary cephalotribe would produce less grave and dangerous injuries to the maternal parts. This conviction, however, should not lead to rash action; in every doubtful case plenty of time should be allowed to the natural forces, and the forceps even may be cautiously tried before resorting to symphyseotomy.

In the employment of version in cases of moderate contraction,

Morisani has no confidence. The mortality among the children is enormous ; Zweifel puts it at 29 per cent.

Two or three combinations of other operations with symphyseotomy are discussed ; the combination with induction of premature labor is rejected ; the combination with embryotomy in certain rare cases is considered advantageous.

The operative details are easy of execution, and do not involve any danger to the patient worth mentioning. The curved bistoury of Galbiati is not essential. Any knife with a protected point and a narrow, strong blade will do. Morisani has never met with ossification of the symphysis, but admits that a chain saw may be required when there is great obliquity and irregularity of the symphysis. It is essential that the accoucheur ascertain that the subpubic ligament is completely divided, and that all the structures of the joint are so cut as not to interfere with the separation of the surfaces. When the thighs are abducted, the separation should extend at once to 4 or 5 cm., and in some cases even to 6 or 7 cm. As soon as this separation has taken place Morisani applies the forceps and completes the labor. He justifies this course as against Zweifel's advocacy of delay. When the labor is over, the wound is at once closed with silk sutures, which are applied as both deep and superficial. Osseous suture is shown by Morisani's statistics to be unnecessary. He has also given up special appliances for holding the pelvis immovable. Simply bandaging and tying the knees together suffice for obtaining a perfect cure.

Hemorrhage has never been a troublesome feature in Morisani's operation. *Tamponnement* of the wound has always been sufficient to arrest it. The lacerations of the anterior vaginal wall, spoken of by some, have figured little in the practice of the operators at Naples. They might be altogether avoided if primiparæ were not operated on, and if the innominate bones were pushed together towards the end of the extraction so as to support the soft parts.

In comparing symphyseotomy with other operations, Morisani thinks that it does not come into competition with Cesarean section, inasmuch as one begins where the other leaves off. With a conjugate diameter of less than 6.7 cm., the Cesarean operation "conserves its rights." He deprecates the resort to abdominal section, with its mortality of 12 per cent., in cases where symphyseotomy should give a favorable result.

As to craniotomy on the living child, the maternal mortality is appreciable, and the balance of advantage lies with symphyseotomy. As to the advantages of induction of premature labor, Morisani has changed his mind. If the deformity of the pelvis be such as to require, for the birth of a living child, induction at the end of the seventh month, or in the course of the eighth, then symphyseotomy should be resorted to. The results of induction so early are "deplorable." "The great majority of the children die," in spite of all the appliances to keep them alive.

The statistics of symphyseotomy appear to require careful handling. The number of cases collected from various sources is 241, showing a maternal mortality of 11.6 per cent. Some of the deaths were due to causes unconnected with the operation. The result of the 241 operations, as far as the fetus was concerned, was 186 living children and 55 deaths. Of these deaths it is sought to explain away 25.

Morisani's own results, as published in January, 1893, in the paper already referred to, show 55 cases in the five years from March, 1887, with two mothers and five children lost, a very remarkable maternal mortality for such an operation.

Morisani's final summary does not contain any fresh point, except, perhaps, his insistence upon the disastrous results of operating upon women with pelves contracted beyond the minimum of 6.7 cm. of conjugate diameter.—*Medical Chronicle*, Sept., 1894.

